# Knowledge, Belief and Practice of Cervical Cancer Screening and Prevention among Women of Taraba, North-East Nigeria

**DOI:** 10.31557/APJCP.2019.20.11.3291

**Published:** 2019

**Authors:** Rosethe Rimande-Joel, Golda Obiageri Ekenedo

**Affiliations:** *Department of Human Kinetics and Health Education, University of Port Harcourt, Nigeria. *

**Keywords:** Cervical cancer, knowledge, beliefs, screening, prevention

## Abstract

**Objective:**

The rising prevalence of cancer of the cervix especially in developing countries gives cause for concern. Fortunately, it can be prevented especially when the women at risk possess correct knowledge, have the right belief and access to screening and prevention services. Previous studies have reported poor knowledge and screening practices among women in developing countries raising the fear of continued spread. The aim of the study was to establish the cervical cancer knowledge, belief and prevention/screening practices among women in Taraba, North-East Nigeria.

**Methods:**

The study adopted a cross sectional survey design. A self-designed structured and validated questionnaire with a reliability index of .82 was employed to elicit information from 978 women of child bearing age. Data collected were analysed using percentage, mean, Chi-Square and ANOVA statistics.

**Result:**

The findings of the study revealed that the women of Taraba had appropriate knowledge about the nature of cervical cancer (73. 2%) signs and symptoms of cervical cancer (76.5%) risk factors of cervical cancer (88.0%), and prevention of cervical cancer (90.3%). The women did not have the right beliefs about the concept of cervical cancer and its preventive measures. They believed that the screening procedure is painful; they were too young to contract the disease and risk stigmatization if they went for screening. Only 45.2% of the women regularly engaged in screening and other prevention practices. Location of residence, and religion significantly determined knowledge and screening/prevention practices (P<0.05) while marital status and age (P<0.05) significantly affected the respondents’ beliefs about cervical cancer and prevention practices. Location of residence, and religion significantly determined knowledge and screening/prevention practices (P<0.05) while marital status and age (P<0.05) significantly affected the respondents’ beliefs about cervical cancer and prevention practices.

**Conclusion:**

It was concluded that knowledge about cervical cancer did not translate to right belief and good practice, and that belief and practice were affected by demographic variables of location of residence, religion, marital status and age.

## Introduction

Cancer of the cervix otherwise known as Cervical Cancer is a disease of public health importance affecting a significant number of women globally. Cancer of the cervix is the commonest female genital tract growth and has been ranked the second most common cancer affecting women globally with developing countries accounting for 85% of the 1.5 million cases of clinically recognized cases (Ferlay et al., 2012). Analysis of the burden of the disease in Northern Nigeria where Taraba State is also located showed that overall cost of illness ranged from US$524 to US$2,743 for local to metastatic diseases respectively covering medical costs and productivity costs (Akinfenwa and Monsur, 2018). Based on the analysis, it was estimated that it would be 16 to 902 times cheaper to screen for the disease than to treat.

It is sad to note that while incidence of cervical cancer has been reduced by 70% in industrialized countries in the last 50 years, the burden seems to be on the increase in developing countries with further rise in the occurrence anticipated from 444,546 to 588,922 between 2012 and 2025 (WHO, 2012). Approximately 90% of deaths from cervical cancer occurred in low and middle income countries with Nigeria having the highest death rate in the whole of Africa (WHO, 2019). About 14,089 women are affected every year out of which 8,240 lost their lives, and it is projected that in 2025 Nigeria will record 19,440 new cases of the disease and 10,991 deaths WHO (2012). 

The ugly statistics regarding cervical cancer morbidity and mortality notwithstanding, the disease can be tackled and brought under control through programmes such as prevention, early diagnosis, and effective screening programmes (WHO, 2019). Cervical cancer can be treated successfully if detected early. Screening for cervical cancer is, therefore, one of the basic preventive measures for the disease, however, access to screening services has been a major problem for women in developing countries especially those in the rural areas. WHO/ICO (2010) had recorded that of about 40.43 million women at risk of developing cervical cancer, 60% are rural dwellers with no access to screening services. Less than 10% of women in Nigeria have had cervical screening as against 40% – 50% of women who are screened in developed countries (Curado et al., 2007). Therefore, most women with the disease present late to hospitals at the invasive stage of the disease when therapy will only result in partial cure or no cure at all (Oguntayo et al., 2015). The current situation is indeed frightening and deserves urgent intervention. Unfortunately, Nigeria lacks a well-articulated extensively circulated nationwide cervical cancer policy and a nation-wide cancer screening programme (WHO, 2012), infrastructure, well trained health staff and financial strength (Toye et al., 2017) to pursue cervical cancer prevention.

A number of factors have been attributed to the high cervical cancer burden in Africa including poor health-seeking behaviour; poor screening strategy, planning and implementation; and lack of political, financial, logistic, and intellectual commitment by all stakeholders (WHO, 2009). Of all these, poor health-seeking behaviour is the only factor within the control of the women, the rest are the responsibilities of government and other institutions. Health seeking behaviour is the action taken by an individual in the event of perceived signs and symptoms or in a bid to prevent the occurrence of disease. Interestingly, such behaviour can be greatly influenced by factors such as knowledge and belief held about the health problem. Though it has been argued that knowledge per se does not guarantee positive health behaviour, yet one can argue that individuals will do only by accident that which they possess no knowledge about.

A number of theories and models have emerged over the years in a bid to understand health behaviours and factors that influence them. The present study anchors on one of such models, the Health Belief Model (HBM) in order to understand the cervical cancer screening and prevention behaviours among women of Taraba, North-East Nigeria. HBM which was developed in the 1990s by Hochbaum, Rosenstock and Kegels has been used to predict a wide range of health-related behaviours including screening for early detection of diseases.

The basic assumptions of the HBM are that people’s beliefs about whether or not they were susceptible to disease, and their perceptions of the benefits of trying to avoid it, influenced their readiness to act. The HBM has four major constructs namely: perceived susceptibility, perceived severity, perceived benefits, and perceived barriers; and three additional constructs which are modifying variables, cues to action and self-efficacy. Perceived susceptibility is an individual’s appraisal of the risk of developing a health problem, (Glanzet al., 2008). The model assumes that a person who perceives the likelihood of being at risk to develop a disease will engage in behaviors to decrease risk. Hence, the screening and prevention practices of the women of Taraba towards cervical cancer will most probably depend on their perception of their susceptibility to cervical cancer.

Again, the second construct, perceived severity, which describes an individual’s assessment of the seriousness of developing a health problem suggests that individuals are likely to take on behaviours that will prevent health problem or reduce its severity if they perceive a particular health problem as serious. The construct includes beliefs as to whether the disease poses a threat to life or could result in disability or cause pain. For example, believing that infection with HPV can lead to cervical cancer, give pain or even cause death is likely to make the women of Taraba take actions to prevent cervical cancer and reduce its severity.

A perceived benefit explains the assessment individuals make regarding the value of getting involved in healthy behaviour to reduce the risk or severity of the disease. This means that believing that taking action would reduce their susceptibility to the condition or its severity can spur the women to engage in healthy behavior like participating in cervical cancer screening to know their status, commencement of early treatment and avoiding multiple sexual partners.

A fourth construct of the HBM that is of significance to this study is modifying variables. Modifying variables are variables that affect health-related behaviours; indirectly by affecting perceived susceptibility, seriousness, benefits, and barriers (Glanz et al., 2008). Modifying variables include: demographic, psychosocial and structural variables; Demographic variables include age, sex, race, ethnicity, and education among others; Psychosocial variables include personality, social class, and peer and reference group pressure; while structural variables include factors such as knowledge about a given disease and prior contact with the disease (Rosenstock and Irwin, 1974). Hence, accurate and sufficient knowledge about cervical cancer, positive belief and attitude towards the disease would contribute to the uptake of cervical cancer screening and prevention practices. On the other hand, the negative effect of these variables will hinder the uptake of preventive measures. 

Empirical studies on cervical cancer screening have suggested that women lack sufficient information on cervical cancer screening (Abotchieand Shokar, 2009; Adanu, 2010); hence, lack awareness and knowledge of cervical cancer, its prevention and screening (Mupepi et al., 2011; Sampselle and Johnson, 2011; Adamu et al., 2012; Getahun et al., 2013; Jansirani et al., 2014; Abiodunet al., 2014; Ebu et al., 2015). 

Since knowledge and belief held about a disease play significant roles in predicting if the individual would adopt positive health behaviour towards treatment and prevention of the disease, the researchers considered it pertinent to find out the knowledge and belief of women of Taraba in North-East Nigeria about cervical cancer. It was considered equally important to establish the prevailing practices of the women with regards to cervical cancer screening and prevention as this would serve to identify intervention needs. Furthermore, as established by the HBM, this study believes that demographic variables can affect cervical cancer screening and prevention behaviour directly and indirectly by affecting knowledge and belief about cervical cancer. 

The purpose of this study, therefore, was to determine the knowledge, belief about cervical cancer and practice of cervical cancer screening and prevention among women of Taraba, North-East Nigeria. A further purpose of this study was to ascertain the association between demographic variables of educational status, marital status, age, religion and location of the residence on the women’s knowledge, belief and practice of cervical cancer screening and prevention.

## Materials and Methods

The study adopted a cross-sectional survey design. The design was successfully utilized by Jansirani, Bhuvaneshwari and Kuberan (2014) to study knowledge, awareness and prevention of cervical cancer among women; attending a tertiary care hospital in Puducherry, India. The sample for the study consisted of 1,200 women residing in Taraba State, North-East Nigeria who were aged 15 to 49 at the time of the study. These women are in their reproductive age and are sexually active placing them at higher risk for HPV infection. Moreover, WHO (2019) identified women 30 – 49 years as target group for cervical cancer screening. The sample size was determined using Taro Yamane formula (Yamane, 1973), and respondents selected through a multi-stage sampling procedure involving non-proportionate stratified sampling, simple random sampling, purposive sampling and accidental sampling techniques. First, the area of the study was stratified into urban, semi-urban and rural areas. Thereafter, simple random sampling was employed in selecting two Local Government Areas (LGAs): one semi-urban out of existing two and one rural LGA out of existing three. The only urban LGA was purposively selected. The next stage was the use of a non-proportionate sampling technique to select four hundred (400) respondents from each LGA making a total of one thousand two hundred (1,200). The respondents were picked from all the government health facilities and institutions in the sampled LGAs using accidental sampling technique. 

The instrument used in collecting data was a self-structured and validated questionnaire titled “Cervical Cancer Knowledge, Belief and Prevention Practices Questionnaire (CCAKPPQ)” with a reliability index of 0.82. The instrument has two sections: A and B. Section A contained demographic information of the respondents while Section B gathered information on the dependent variables of knowledge, belief and practice of cervical cancer screening and prevention. The section contains 24 knowledge items with response options of True/False. Belief about cervical cancer screening and prevention was measured with 16 item-modified Likert-type scale of Strongly Agree (4pts), Agree (3pts), Disagree (2pts) and Strongly Disagree (1pt). Nine questionnaire items with response options of Always, Occasionally and Never was used to measure cervical cancer prevention practices. The reliability of the instrument was established through a pilot study. Data collected was analysed using Pearson Product Moment correlation and Cronbach alpha. The correlation yielded coefficients of 0.86, 0.77 and 0.83 for knowledge, belief and practice respectively. The overall reliability index was 0.82.

Consent form was attached to copies of the questionnaire which were distributed to the respondents. The content of the form and the questionnaire were translated into local languages and dialects for illiterate and semi-literate respondents through the instrumentality of four research assistants. Out of the 1,200 copies of the questionnaire distributed, 978 copies were duly filled and returned given a return rate of 81.5%. Data collected were analyzed using the Statistical Package for Social Sciences – SPSS version 21. Percentage was used to answer the research questions on knowledge and practice of cervical cancer screening and prevention while mean was used to answer the research question on belief about cervical cancer. Chi-square was used to test hypotheses of no significant difference in knowledge and practice of cervical cancer screening and prevention, while Analysis of Variance (ANOVA) and t-test were used to test the hypotheses of no significant difference in belief among the women all at 0.05 alpha levels. A criterion mean of 2.5 was used to decide on whether the belief is positive or negative. A mean of 2.5 and above was regarded as a positive belief while a mean of below 2.5 was regarded as negative belief.

## Results

Table 1 shows that majority of the respondents possessed knowledge about the nature of cervical cancer (73. 2%) signs and symptoms of cervical cancer (76.5%) risk factors of cervical cancer (88.0%), and prevention of cervical cancer (90.3%) with an overall percentage of 82.2. Data in table 1further show that the respondents generally had a negative belief about cervical cancer with a grand mean of 2.45 (< crit $\bar{X}$ =2.50). Specifically, the respondents had negative belief about not developing cervical cancer because they were young ( $\bar{X}$ = 1.02), going for screening being a lack of faith in God ( $\bar{X}$ = 2.19), the screening process being painful ( $\bar{X}$ = 2.02), being stigmatized if their screening comes out positive ( $\bar{X}$ = 2.21), dying faster if they knew their positive status ( $\bar{X}$ = 2.33), and that cervical cancer affects only married women ( $\bar{X}$ = 2.01). 

More data from Table 1 revealed that overall, the majority (45.2%) of the women always practiced cervical cancer screening/ preventive measures, 25.6% occasionally practised the screening and preventive measures, while 28.3% never observed cervical cancer screening/ preventive measures. Specifically, 94.6%, of them always did not keep multiple sexual partners, 81.2% always went for treatment upon notice of signs and symptoms of sexually transmitted infections, 70% always took measures to avoid contracting sexually transmitted infections, 59.2% always avoided the use of oral contraceptives because of its side effects. Again, while the majority of the respondents (56.9%) occasionally ate fruits and vegetables for prevention of cervical cancer, the majority were never vaccinated against cervical cancer (85.6%), never screened for cervical cancer (67.6%) and never checked their family for the history of cervical cancer (57.8%). 

The results as shown in Table 2 revealed that there was no significant difference in the knowledge of the respondents with regards to their educational status, marital status and age because the calculated values for these variables were less than the table values at df of 3 (educational status: Cal. χ^2^ =5.11<tab. χ^2^ =5.11; marital status: Cal. χ^2^=4.129<tab. χ^2^ =7.82; and age: Cal. χ^2^=4.483<tab. χ^2^ =7.82). Also, the p-values concerning educational status, marital status and age were all greater than 0.05 alpha level (P=0.214, 0.282 and 0.207 respectively). Data in the table show that respondents who lived in a semi-urban area, those who were widowed and those within 16 to 30 years possessed more knowledge about cervical cancer. However, there were significant differences in the knowledge of the respondents about cervical cancer based on location and religion both at df of 2 (location: Cal. χ^2^ =13.915>tab. χ^2^ = 5.99; P=0.036 and religion: Cal. χ^2^ =7.763> tab. χ^2^ =5.99; P=0.013) as shown in the table. Rural dwellers and respondents who were Muslims had the least knowledge about cervical cancer. 

Results in Table 3 indicate that there was statistically significant difference in the respondents’ beliefs about cervical cancer based on marital status (f-cal = 8.358>tab.f =8.53 ) and age (f-cal = 8.348 > tab.f =8.53 ), with p-value of .000, less than α=.05. On the other hand, there was no statistically significant difference in the respondents’ beliefs based on location, educational status, and religion (f-cal = .275, 1.215 and .610 respectively < tab. f=19.50). Also p-values of .760, .303 and .543 respectively were greater than α=.05.

The results as shown in Table 4 revealed that the calculated Chi-value for location and religion (χ^2^ cal = 15.052 and 26.486 respectively) were greater than the table Chi-value of 5.99 at df of 2. Also, the calculated p-values of .005 and .000 respectively for location and religion were less than α=.05, thus indicating that location and religion significantly affected the respondents’ practice of cervical cancer screening and prevention. Rural dwellers and respondents who belonged to religions other than Christianity and Islam had the poorest cervical cancer prevention practices. Muslims and semi-urban dwellers practised cervical cancer prevention more than the others. On the other hand, the calculated Chi-value for educational status, marital status and age (χ^2^ cal = 6.426, 5.347 & 3.240 respectively) were less than the table Chi- value of 7.82 at df of 3. Again, the calculated p-value of 0.377, 0.500 and 0.519 for educational status, marital status and age respectively were greater than α =0.05. Hence, the null hypothesis of no significant difference in cervical cancer screening and prevention practices was accepted for educational status, marital status and age.

**Table 1:** Percentage and Mean Responses on Cervical Cancer Knowledge, Belief, Prevention and Screening Practices of Respondents (N = 978)

Knowledge about cervical cancer	Correct %			Incorrect%		
Knowledge about the nature of cervical cancer	73.2			26.8		
Knowledge of sign and symptoms	76.5			23.5		
Knowledge of risk factors	88.1			11.9		
Knowledge of cervical cancer prevention	90.3			17.9		
Overall %	82.2			17.9		
Beliefs about cervical cancer				Remarks		
I am young and cannot develop cervical cancer	1.03			-ve		
Cervical cancer is a deadly disease	3.20			+ve		
Promiscuity does not predispose to cervical cancer	2.77			+ve		
Cervical cancer is a punishment from the gods	2.65			+ve		
Cervical cancer is caused by witches and wizards	2.71			+ve		
It is embarrassing to undergo cervical pap test.	2.66			+ve		
Going for screening is lack of faith and belief in God	2.19			-ve		
The procedure for cervical cancer screening is painful	2.02			-ve		
If I know my status, I will die before my time	2.33			-ve		
I will be stigmatized if I test positive	2.21			-ve		
I will lose my virginity during the screening	2.58			+ve		
The test is unnecessary and without benefit	2.91			+ve		
Cervical cancer cannot be prevented	2.60			+ve		
Only the gods can prevent it	2.75			+ve		
It is a disease of illiterates	2.57			+ve		
Cervical cancer affects only married women	2.01			-ve		
Grand	2.45			-ve		
Cervical cancer screening and prevention practice	Always		Occasionally		Never	
	f	%	f	%	f	%
I screen for cervical cancer (pap smear).	38	3.9	279	28.5	661	67.6
I examine my vagina for possible abnormality	438	44.8	299	30.6	241	24.6
I eat fruit & vegetables to prevent cervical cancer	317	32.4	556	56.9	105	10.7
I check my family for the history of cervical cancer	91	9.3	322	32.9	565	57.8
I take measures to avoid contracting STI	685	70.0	268	27.4	25	2.6
I do not keep multiple sexual partners	925	94.6	39	4.0	14	1.4
I avoid oral contraceptives due to its side effects	579	49.2	358	36.6	41	4.2
I go for treatment when I notice any sign of STI.	794	81.2	180	18.4	4	0.4
I have been vaccinated against cervical cancer	113	11.6	28	2.9	837	85.6
Overall%	45.2		25.6		283	

**Table 2:** Summary of Chi-Square Analysis of No Significant Difference in the Knowledge about Cervical Cancer among the Women with Regards to the Location of Residence, Educational Status, Marital Status, Age and Religion

Demographic variables	N	Cal.χ^2^	df	tab.χ^2^	p-value	Decision
Location		13.915	2	5.99	0.036	Rejected
Urban	440					
Semi-urban	284					
Rural	254					
Educationalstatus		5.11	3	7.82	0.214	Accepted
Noformal	150					
Primary	184					
Secondary	369					
Tertiary	275					
Maritalstatus		4.129	3	7.82	0.282	Accepted
Single	213					
Married	567					
Divorced	72					
Widowed	126					
Age		4.483	3	7.82	0.207	Accepted
16–30yrs.	364					
31–44yrs.	428					
45yr.+	186					
Religion		7.763	2	5.99	0.013	Rejected
Christianity	513					
Islam	403					
Others	62					

Chi-square two-tailed, α = 0.05

**Table 3:** Summary of ANOVA Analysis of no Significant Difference in Beliefs about Cervical Cancer among the Women with Regards to the Location of Residence, Educational Status, Marital Status, Age and Religion

Demographic variables		Sum of squares	df	Mean square	f-cal	tab. f	p-value	Decision
Location .	B/ G	0.109	2	0.55				
	W/ G	193.405	975	0.198				
	Total	193.51	977		0.275	19.50	0.760	Accepted
								
Educational Status	B/ G	0.721	3	0.240				
	W/ G	192.793	947	0.198				
	Total	193.714	977		1.215	8.53	0.303	Accepted
Marital status	B/ G	4.857	3	1.619				
	W/ G	188.658	947	0.194				
	Total	193.514	977		8.358	8.53	0.000	Rejected
Age	B/ G	3.258	3					
	W/ G	190.256	947	1.629				
	Total	193.514	977	0.195	8.349	8.53	0.000	Rejected
Religion	B/ G	0.242	2					
	W/ G	193.273	975	0.121				
	Total	193.514	977	0.198	0.610	19.50	0.543	Accepted

**Table 4:** Summary of Chi-Square Analysis of No Significant Difference in the Practice of Cervical Cancer Screening and Prevention among the Women with Regards to the Location of Residence, Educational Status, Marital Status, Age and Religion

Demographic variables	N	Cal. χ^2^	df	tab. χ^2^	p-value	Decision
Location		15.052	2	5.99	0.005	Rejected
Urban	440					
Semi-urban	284					
Rural	254					
Educational status		6.426	3	7.82	0.377	Accepted
No formal	150					
Primary	184					
Secondary	369					
Tertiary	275					
Marital status		5.347	3	7.82	0.500	Accepted
Single	213					
Married	567					
Divorced	72					
Widowed	126					
Age		3.240	3	7.82	0.519	Accepted
16 -30yrs	364					
31-44yrs	248					
45yrs +	186					
Religion		26.486	2	5.99	0.000	Rejected
Christianity	513					
Islam	403					
Others	62					

Chi-Square two-tailed, α = 0.05

## Discussion

Knowledge has been identified as critical to the prevention and control of cervical cancer. The findings of this study which show that majority of the respondents had appropriate knowledge about cervical cancer is a pleasant surprise considering that similar studies in Nigeria and other sub-Saharan Africa made contrary findings. For instance, Ebu et al., (2015) found that in Ghana, 68.4% of women studied had no awareness of the disease, 93.2% had no knowledge of the risk factors and 97.7% never heard of a pap smear. Similarly, Abiodun, Fatungase et al., (2014) discovered that 93.3% of women from Ogun State, Western part of Nigeria had poor knowledge about cervical cancer. The situation was the same in India where Jansirani et al., (2014) found poor knowledge and perception about cervical cancer among women attending antenatal care. This finding points to the fact that a lot of enlightenment concerning cervical cancer may have been going on in Taraba State. Part of the reason for this impressive result could be the reported sensitization campaign on cervical cancer carried out by the wife of the Governor of Taraba State in 2015 which featured health talks on diseases associated with women. It was also gathered during fieldwork from the nurses that women were receiving health information about cervical cancer during ante-natal health education in Government-owned health facilities. This finding is encouraging because possession of knowledge portends hope for positive belief, attitude and improved cervical cancer prevention practices among the women, and government and non-governmental agencies can leverage on such baseline data for future interventions.

Despite the possession of appropriate knowledge, the women generally did not have the right beliefs about the concept of cervical cancer and its preventive measures. They believed that they were too young to contract the disease and risk stigmatization if they went for screening. They also believed the screening processes to be a painful one and a show of lack of faith in God. This finding is consistent with that of Ebu et al., (2015) whose respondents believed it was embarrassing and painful to have Pap smear test. This is a dangerous trend because such belief is capable of jeopardizing the essence of early diagnosis and treatment of the disease among the women population in that area. It means that many affected persons are likely to present late to the hospital at an advanced stage and because modern health facilities are lacking in the country only palliative treatment can be given to alleviate pain at this stage pending when the individual finally dies. However, it is soothing to know that the women had the right beliefs concerning the causes of cervical cancer and its preventability. One implication of this finding is that programmes offered in the past may not have factored in the influence of cultural and religious beliefs on health behaviour underscoring the need for proper needs assessment before programmes are designed and implemented. Knowledge programmes should be designed to correct negative beliefs because they can prevent effective transfer of knowledge into practice.

With regards to cervical cancer screening and prevention practices, only 45.2% of the women regularly engaged in screening and other prevention practices while 28.3% never engaged in screening and prevention practices. This is contrary to expectations compared with the level of knowledge displayed earlier. This odd situation may be accounted for by the wrong beliefs held by the women about their vulnerability to cervical cancer and about cervical cancer screening. By this finding, it can be estimated that 54.8% of the population was vulnerable and at risk of developing cervical cancer; since 85.6% never had vaccination and 54.8% were not seriously engaged in screening and prevention practices. These findings agreed with Ezem (2007), and Ahmed, Sabitu, Idris, and Ahmed et al., (2013) who documented very poor uptake of screening irrespective of respondents’ possession of good knowledge.

Some demographic variables played significant roles in shaping knowledge, beliefs and screening/preventive practices of the women. Table 2 shows that location of residence with regards to urban and rural, and religion were factors found to be associated with the women’s knowledge about cervical cancer. Rural women had less knowledge which is not surprising since oftentimes rural areas are not adequately covered with government programmes due mainly to barriers such as lack of social infrastructures like electricity, information and communication technology, access roads and language barriers. Such neglect is at the expense of the rural women’s chances of getting the needed services for cervical cancer prevention and may also explain why location was also significantly associated with women’s screening and prevention practices. They may not have easy access to screening services in the way that those in urban and semi-urban areas would.

Religion in most African culture is a strong determinant of behaviour. Though both major religions in Taraba, Christianity and Islam are conservative in their approach to issues, Islam appears to be more stringent in enforcing their doctrines some of which may act as limiting factors to the women’s access to health care services including health education. Hence, it is not surprising that Moslem women had less knowledge about cervical cancer, screening and prevention. On the other hand, Moslem women were found to have better prevention practices than women of other religions. 

Surprisingly, religion did not significantly influence the women’s beliefs about cervical cancer, rather marital status and age did. Perhaps, experience played a significant role here since marriage and age brings about experience and maturity which help in shaping peoples beliefs. Younger women dangerously believed they could not get the disease because they were young. This assumption is consistent with a study which reported that a greater part of the participants believed that older women are at greater risk of having cervical cancer (Barron and Houfek, 2001). However, it is essential to target this younger group because they are sexually active and the chance of getting exposed to human Papillomavirus is high. This calls for education interventions in schools and existing curriculum on reproductive health expanded to accommodate more essential information on cervical cancer. Knowing about the disease earlier will help them have the right beliefs, attitudes and prevention practices.

Another significant and surprising finding of this study is that no significant difference was found in the knowledge, beliefs and practices of the women based on their educational status. One would have reasoned that education should provide better leverage for increased knowledge and more appropriate beliefs and screening/prevention practices concerning cervical cancer. It can, therefore, be said that cervical cancer education programmes were effective in reaching women of different educational and social backgrounds.

Based on the findings of the study, the researchers concluded that knowledge about cervical cancer among women of Taraba did not exactly translate to the right beliefs and positive screening/prevention practices. Wrong beliefs and poor screening and prevention practices could be attributed to factors such as the location of residence, religion, marital status and age. More active involvement by the women in cervical cancer screening and prevention activities will be achieved if concerned agencies within and outside the government device workable means of reaching the rural communities with screening and prevention programmes as well as deliver culture-sensitive programmes.

## Statement on conflict of interest

There is no conflict of interest.

## Ethical approval

Ethical approval for the study was obtained from the Ethical Committee of Taraba State Specialist Hospital, Jalingo. 
